# Analysis of targeted somatic mutations in pleomorphic carcinoma of the lung using next‐generation sequencing technique

**DOI:** 10.1111/1759-7714.13536

**Published:** 2020-06-23

**Authors:** Saki Manabe, Rika Kasajima, Shuji Murakami, Yohei Miyagi, Tomoyuki Yokose, Tetsuro Kondo, Haruhiro Saito, Hiroyuki Ito, Takeshi Kaneko, Kouzo Yamada

**Affiliations:** ^1^ Department of Thoracic Oncology Kanagawa Cancer Center Yokohama Japan; ^2^ Molecular Pathology and Genetics Division Kanagawa Cancer Center Research Institute Yokohama Japan; ^3^ Department of Pathology Kanagawa Cancer Center Yokohama Japan; ^4^ Department of Pulmonology Yokohama City University Graduate School of Medicine Yokohama Japan

**Keywords:** Comutation, next‐generation sequencing, pleomorphic carcinoma, *TP53* mutation

## Abstract

**Background:**

Pleomorphic carcinoma (PC) of the lung is a rare type of lung cancer with aggressive characteristics and a poor prognosis. Because it is rare, the molecular characteristics of PC remain unclear.

**Methods:**

A gene mutation analysis was performed using next‐generation sequencing (NGS) in patients with PC of the lung who had undergone surgical resection.

**Results:**

A total of nine patients were enrolled in the study. All the patients were male and eight had a history of smoking. Eight tumors contained spindle cells and three contained giant cells. Mutations considered significant were found in eight of the nine patients: in *TP53* in five patients, in *MET* in two patients, and in *ALK*, *ERBB2*, *PIK3CA*, *APC*, *NF1*, and *CDKN2A* in one patient each. No *EGFR* mutation was detected in our analysis. Co‐mutations were detected in three patients: *TP53* with *MET* and *NF1*, *TP53 with ERBB2*, and *PIK3CA* with *CDKN2A*.

**Conclusions:**

*TP53* mutations were detected most frequently in PC of the lung with NGS analysis. Different co‐mutations were seen in several specimens.

**Key points:**

- Significant findings of the study
  This study demonstrates that mutations in the TP53 gene are frequently found and co‐mutations are sometimes found in pleomorphic carcinoma of the lung using genomic profiling analysis.
- What this study adds
  Our results will help to analogize the genetic characteristics and potential target of molecular‐targeted agents of pleomorphic carcinoma of the lung.

## Introduction

Lung cancer is the leading cause of cancer death worldwide^1^ and well‐known risk factors for lung cancer include smoking and some chemicals.^2^, ^3^ Gene mutations linked to carcinogenesis have been identified in the past decade, some of which strongly accelerate cancer growth and progression, and are called driver‐gene mutations. Research into non‐small cell lung cancer (NSCLC) has resulted in the development of several molecular therapeutic agents that target driver mutations, such as those in epidermal growth factor receptor (*EGFR*),^4^, ^5^, ^6^ anaplastic lymphoma kinase (*ALK*),^7^, ^8^ *ROS1*,^9^, ^10^ and *BRAF*.^11^ These have a greater tumor‐shrinking effect and lower toxicity and confer longer progression‐free survival (PFS) than conventional cytotoxic drugs. In contrast, lung cancers with other gene mutations, such as in *TP53*, have shown shorter overall survival (OS), regardless of their driver mutation status.^12^ Therefore, genomic information on individual tumors is important to guide the selection of rational therapeutic strategies with which to improve the outcomes of patients with advanced NSCLC. Recently developed techniques to identify specific gene changes have accelerated the trend towards personalized therapies. PCR‐based testing, immunohistochemistry (IHC), and fluorescence in situ hybridization are routinely used to detect driver mutations, and next‐generation sequencing (NGS) has recently become a key technique for molecular screening. However, a personalized approach based on specific oncogenic variations is particularly useful in lung adenocarcinoma and several other pathological types of lung cancer.

Pleomorphic carcinoma (PC) of the lung accounts for 0.9%–1.6% of all primary lung cancers.^13^, ^14^, ^15^, ^16^ PC of the lung is a heterogeneous group of NSCLCs that contain at least 10% spindle and/or giant cells and carcinomas that consist of only spindle and giant cells.^17^ PC of the lung is known to be highly progressive and less susceptible to cytotoxic anticancer agents.^18^, ^19^, ^20^ The median PFS is only 1.5 months and the median OS is 7.2 months in patients with PC of the lung treated with platinum‐based chemotherapy.^20^ Even in surgically‐resected patients, the median OS is about 14.7 months.^21^ Therefore, the development of new treatment strategies is urgently required.

A few cases of pulmonary sarcomatoid carcinoma (PSC), which according to the pathological classification of the World Health Organization includes PC, that carry specific gene mutations have been reported.^22^, ^23^ However, the frequency of driver mutations in PC of the lung is still unknown. There may be several genetic factors that have a poor prognosis in patients with PC of the lung, and the detection of gene mutations carried by PC may clarify the cause of its rare pathological morphology, the mechanism of its treatment resistance, and targets for new treatment strategies. The aim of this study was to analyze the predictive factors for a poor outcome and potential targets of targeted therapeutic agents by determining genetic variation profiles of PC of the lung with NGS analyses.

## Methods

### Study population

We reviewed the records of 944 patients with primary lung cancer who had undergone surgical resection between 2007 and 2014 at Kanagawa Cancer Center, Yokohama, Japan, and extracted 21 specimens that had been diagnosed as PC. Nine of the 21 specimens, which had been stored as frozen tumor materials, were recruited for our study. All nine tumors were reviewed and diagnosed by two pathologists (Y.M. and T.Y.), according to the 2015 World Health Organization (WHO) classification of lung tumors.^17^

All clinical information, such as patient age, sex, pathological stage, and smoking status, were obtained from the medical records. The study was approved by the Institutional Review Board of Kanagawa Cancer Center (approval No. Eki‐73‐2015), and written informed consent was obtained from each patient.

### Design of original Ion AmpliSeq DNA panels for the detection of genetic changes

With reference to several studies that comprehensively examined genetic changes in tumors, we constructed an original gene cancer panel to detect genetic changes that have been reported at high frequencies in adenocarcinomas of the lung.^24^, ^25^, ^26^, ^27^ We then chose 41 genes that were frequently detected in these tumors and were considered to be biologically significant, to construct an original cancer gene panel, designated ‘KCC41’ (Table S1).^28^

### Genomic DNA and RNA extraction

To obtain tumor DNA and RNA samples, first, we prepared a thinly sliced section from each stored frozen specimen, stained with hematoxylin and eosin (HE), and confirmed morphologically that each tissue actually contained tumor cells. DNA and RNA were then extracted from the frozen tissues with the ZR‐Duet DNA/RNA Miniprep Kit (Zymo Research, Irvine, CA, USA), according to the manufacturer's instructions. DNA was also extracted from formalin‐fixed paraffin‐embedded (FFPE) tissues with NucleoSpin DNA FFPE (Macherey‐Nagel, Düren, Germany), according to the manufacturer's instructions.

### Quantity and quality checks of DNA and RNA

DNA and RNA were quantified with Qubit 2 (Thermo Fisher Scientific, Waltham, MA, USA). The DNA and RNA quality was assessed with the optical density ratios A_260_/A_280_ and A_260_/A_230_, respectively, using a NanoPhotometer (Implen). Genomic DNA (10 ng) or total RNA (10 ng) was used to create panel libraries for each specimen. The degradation of the DNA extracts from the FFPE tissues was estimated with a TaqMan PCR assay using TaqMan RNase P Detection Reagents Kit (short assay: 87 bp) (Thermo Fisher Scientific) and TaqMan MGB gene expression detection kit for the FFPE DNA QC assay (long assay: 256 bp) (Thermo Fisher Scientific). The DNA samples for which the relative value of the long assay/short assay (Ct value) exceeded 0.2 were selected for the panel analysis.

### Library preparation and sequencing with the original DNA panel, commercial DNA, and RNA panel

In this study, we used three types of panels: the KCC41 panel, the Cancer Hotspot Panel v2 (CHPv2) (Thermo Fisher Scientific), and the Ion AmpliSeq RNA Fusion Lung Cancer Research Panel (Thermo Fisher Scientific). Genomic DNA (10 ng) or total RNA (10 ng) was used to create the panel libraries for each specimen. The libraries were amplified with the Ion Torrent AmpliSeq technology, and sequenced with an Ion PGM System for Next‐Generation Sequencing (Thermo Fisher Scientific). Each pooled library was quantified with an Agilent High Sensitivity DNA Kit (Agilent Technologies, USA) with an Agilent 2100 Bioanalyzer (Agilent Technologies). Emulsion PCRs were performed with libraries diluted to 100 pM to create template‐positive Ion Sphere Particles (ISPs) using the OneTouch 2 (OT2) instrument (Thermo Fisher Scientific) and the Ion PGM Template OT2 200 Kit (Thermo Fisher Scientific). The template‐positive ISPs were enriched with Ion OneTouch ES (Thermo Fisher Scientific). The Ion PGM 200 Sequencing Kit was used for sequencing.

### Variant call and validation

The Torrent Suite v4.0.2 and Ion Reporter version 4.4 software (Thermo Fisher Scientific) were used to process and analyze the Ion PGM sequence data. Quality control reports were obtained with the Torrent Suite. Single‐nucleotide variants (SNVs) and insertions/deletions (indels) with a coverage of ≥20 reads that encoded amino acid changes relative to the UCSC hg19 reference genome sequence were selected to identify somatic variants. Single‐nucleotide polymorphisms (SNPs) were then excluded by using the sequences as queries against the databases COSMIC (http://cancer.sanger.ac.uk/cosmic), dbSNPs (NCBI, NIH; https://www.ncbi.nlm.nih.gov/projects/SNP/), the 1000 Genomes Project (http://www.1000genomes.org), cBioPortal (https://www.cbioportal.org), and other publicly accessible databases. If the database analysis failed to clarify whether an SNV was an SNP or a somatic variant, Sanger sequencing of DNA from a non‐neoplastic counterpart of each specimen was performed to obtain a definitive result. Finally, sequence changes with an allele frequency of >2% were defined as somatic variants.

### Detection of fusion transcripts and *ALK* allelic imbalance

Torrent Suite v4.0.2 and Ion Reporter version 4.4 were used to process and analyze the sequence data from the Ion AmpliSe RNA Fusion Lung Cancer Research Panel (Thermo Fisher Scientific). Quality control reports were obtained with the Torrent Suite server. The Ion AmpliSeq RNA Fusion Lung Cancer Research Panel is based on an amplicon target enrichment approach that allows the amplification and detection of 70 known fusion transcripts of the *ALK*, *RET*, *ROS1*, and *NTRK1* genes, using a pair of primers specific for each fusion (Table S2). The 3′/5′ ratios are calculated for the four genes included in the panel. If no common fusion transcripts are detected, an imbalanced ratio may reflect the presence of fusion transcripts in the sample.

### Immunohistochemistry for ALK protein expression

Thin‐sliced FFPE tissues were prepared on silane‐coated slides for immunohistochemistry (IHC). We used the highly sensitive N‐Histofine ALK Detection Kit (Nichirei Bioscience, Tokyo, Japan), which is based on the intercalated antibody‐enhanced polymer method and includes clone 5A4 as the anti‐ALK primary antibody.^29^, ^30^

A semiquantitative assessment was made by estimating the percentage of IHC‐positive tumor cells. ALK‐IHC scoring was as follows: 0 = no stained cells; 1 = 0%–50% stained tumor cells; 2 = 51%–80% stained tumor cells; 3 = >80% stained tumor cells. A score ≥ 1 was deemed to be positive.

## Results

### Patient characteristics

A total of nine patients pathologically diagnosed with PC, according to the WHO classification, were enrolled in the study. Table 1 provides the patients' characteristics, including age, sex, pathological subtype of the resected tissue, staging, and smoking status. The median age was 69 years (range, 44–78 years), all patients were male, and eight patients had a smoking history. Eight tumors contained spindle cells and three contained giant cells.

**Table 1:** Clinical features of nine patients with pleomorphic carcinoma of the lung

			Pathological features			
No.	Age	Sex	Epithelial component	Sarcomatoid component	p‐stage	Smoking status (Pack‐years)
1	57	M	Adeno	Spindle cell, Giant cell	IIB	32
2	71	M	Squamous	Spindle cell	IIIA	72
3	67	M	Squamous	Spindle cell	IB	90
4	77	M	Large	Spindle cell	IB	52
5	78	M	Adeno	Spindle cell	IIA	64
6	73	M	Adeno	Spindle cell, Giant cell	IIB	104
7	69	M	Adeno	Spindle cell	IIB	51
8	54	M	Large	Giant cell	IIB	41
9	44	M	Adeno	Spindle cell	IIIA	0

M, male; p‐stage, pathological stage; Adeno, adenocarcinoma; Squamous, squamous cell carcinoma; Large, large cell carcinoma.

### Gene variants identified with KCC41 and CHPv2 panel analyses

**Figure 1:**
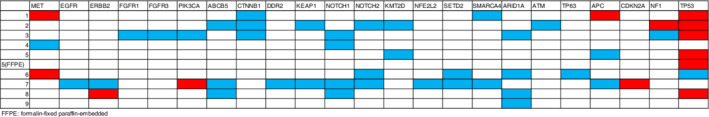
Mutational summary of nine patients with pleomorphic carcinoma. Genes in red were confirmed as pathogenic variants and genes in blue were considered variants of uncertain or unknown significance (VUSs).

The DNA sequencing results from the KCC41 panel are shown in Figure 1. Because the tumor content of patient No. 5 was low (about 10%), we performed an additional panel analysis with the Cancer Hotspot Panel version 2. The confirmed somatic nucleotide sequence changes in nonsynonymous SNVs and indels, with or without frameshifts, were considered to be somatic variants in this study. Somatic variants were detected in all the patients with our panel analyses. A total of 58 somatic variants were identified in 23 of the 41 lung‐adenocarcinoma‐related genes in the KCC41 panel. The results are summarized in Figure 1 (detailed information is provided in Table S3). A total of 11 probable pathogenic variants in six genes were identified in the present KCC41 panel analysis. A known driver‐gene variant reported in the literature was detected in *TP53* (p.R175H).^31^ Although not well characterized as driver genes in the literature, somatic variants with high pathogenic scores predicted with Functional Analysis through Hidden Markov Models (FATHMM) (http://fathmm.biocompute.org.uk) were also identified in *TP53* (p.H178D, p.R175H, p.H193Y, p.R249M, and p.C242S), *PI3KCA* (p.R357L), *NF1* (p.R304*), *APC* (p.E1209*), *MET* (p.L211W, p.H1112Y), and *ERBB2* (p.S1050L). Although a FATHMM score was not assigned to *CDKN2A* p.L120*, this causes the premature termination of a tumor suppressor protein and we consider *CDKN2A* (p.E120*) to be a pathogenic variant. The remaining two variants were considered to be variants of uncertain or unknown significance (VUSs). These variants were found in the COSMIC v90 database, with labels of “n/a” or “neutral” based on their FATHMM scores. The remaining 44 somatic mutations were not recorded in COSMIC.

In summary, eight of the nine patients showed more than one gene mutation considered to be pathogenic, and five patients showed a mutation in *TP53*. Combinations of gene mutations considered to be pathogenic were detected in three patients: two patients had two mutations (in *PIK3CA* and *CDKN2A* or in *ERBB2* and *TP53*) and one patient had three mutations (in *MET*, *APC*, and *TP53*).

### ALK‐IHC and fusion transcript analysis with the Ion AmpliSeq RNA fusion lung research panel

The results of the *ALK* mutation analysis are provided in Table 2. ALK‐IHC showed focal staining in patient No. 7 and diffuse staining in patient No. 9. In addition to IHC, we analyzed ALK fusion transcripts with the Ion AmpliSeq RNA Fusion Lung Research Panel, and demonstrated *EML4* (E6)–*ALK* (E20) fusion transcripts in patient No. 9, but no common ALK fusion transcripts in patient No. 7. The allelic imbalance in the *ALK* gene was calculated and no ALK fusion was suggested in patient No. 7. Therefore, *ALK* mutation was only detected in patient No. 9, and patient No. 7 was negative for ALK fusion, although ALK‐IHC was positive in this patient.

**Table 2:** Results of ALK immunohistochemistry (IHC), ALK fusion detection with sequencing, and an allelic imbalance analysis

No.	1	2	3	4	5	6	7	8	9
ALK‐IHC (iScore*)	0	0	0	0	0	0	1	0	3
ALK‐fusion	−	−	−	−	−	−	−	−	EML4(E6)‐ALK(E20)
ALK allelic imbalance	−	−	−	−	−	−	−	−	+

ALK, anaplastic lymphoma kinase; IHC, immunohistochemistry; EML4‐ALK, echinoderm microtubule associated protein like‐4‐anaplastic lymphoma kinase.

## Discussion

PC is a rare pathological type of primary lung cancer, with an aggressive clinical course, that is resistant to cytotoxic chemotherapy.^18^, ^32^ Genetic profiling of PC should be helpful in the selection of treatment strategies, especially for advanced‐stage tumors. However, the pathological diagnosis of PC is generally difficult to substantiate with small biopsy samples. Therefore, we used a genomic profiling analysis of surgical samples from patients diagnosed with PC. There have been few reports of NGS analyses of PC of the lung. Here, we have shown that PC of the lung generally contains many somatic mutations and several driver‐gene mutations. Notably, multiple mutations were seen in some specimens.

Mutations were found in eight (88.9%) of the nine patients in this study, which is higher than expected for the common type of NSCLC.^33^ Moreover, three (33.3%) of the nine patients had multiple mutations. A previous study of PSC demonstrated high gene‐mutation frequencies of 69%–100% and high multiple‐gene‐mutation frequencies of 39.5%–85.7%.^34^, ^35^, ^36^ Conventionally, the driver‐gene mutation usually occurs in an early stage of tumor growth and tumor growth or relapse is only promoted by a single variant,^37^, ^38^ so driver‐gene mutations are considered to be mutually exclusive. PC of the lung includes several histological subtypes, so pathological heterogeneity may affect the co‐mutation profile. In this study, we did not evaluate the mutation types in tumor blocks from different pathological subtypes, but a previous study demonstrated the relative molecular homogeneity of the different histological component in PC of the lung.^36^

Tumorigenesis is known to be related to the loss of suppressor genes, such as *TP53*.^39^ *TP53* is frequently mutated and the TP53 protein inactivated in many types of malignant tumor.^40^, ^41^ In the present study, *TP53* gene mutations were observed in five (55.6%) of the nine patients. This result is similar to previous studies, which demonstrated that the *TP53* gene is mutated in approximately 55% of PSCs.^34^, ^35^ *TP53* is known to be the most frequently co‐mutated gene in all types of lung cancer.^42^ A genome‐wide analysis of *TP53* knockout rats demonstrated that the loss of TP53 is not the main driver of large‐scale structural variations of the genome, but that chromothripsis occurs primarily under TP53‐heterozygous conditions rather than under TP53‐null conditions.^43^ This observation suggests that *TP53* mutations increase genomic instability. *TP53* mutations have also been associated with resistance to chemotherapy and radiation and with a poor prognosis.^44^ PC of the lung generally has a poor prognosis and neither neoadjuvant nor chemotherapy improves patient survival, even in early stage disease.^18^, ^19^, ^21^

The discovery of treatable driver mutations, including in *EGFR* and *ALK*, is associated with sensitivity to molecular targeted therapies and improved clinical outcomes in patients treated with those therapies. However, the frequency of treatable driver mutations in PC of the lung is controversial. The frequency of *EGFR* mutations was 50% in Asian patients with NSCLC.^45^ Several previous studies reported that the frequency of *EGFR* mutations was 0%–23.8% in patients with PC of the lung.^15^, ^46^, ^47^ No *EGFR*‐mutation‐positive PC of the lung was detected in our study. The frequency of EML4–ALK gene fusion is 2%–7% in NSCLC.^48^, ^49^ Several previous studies have reported that the frequency of the EML4–ALK gene fusion is 0–3.5% in patients with PC of the lung or PSC.^20^, ^50^, ^51^, ^52^ Because the frequency of *ALK* rearrangement in NSCLC is low, it is unclear whether it is lower in PC of the lung than in NSCLC. In the present study, ALK‐IHC detected two patients with suspected ALK protein fusion. However, patient No. 7 showed no ALK fusion in an RNA assay and was negative for ALK allelic imbalance, despite focal staining with ALK‐IHC, so we confirmed EML4–ALK gene fusion in only one patient. A comparative analysis of the ALK‐IHC and NGS panel results for fusion transcripts showed high agreement between the two test methods.^53^ False‐positive ALK‐IHC results are possible in nonadenocarcinomas, although not in adenocarcinomas.^29^ The fusion panel is theoretically the most reliable assay for the detection of mutant transcripts because it supplies direct evidence of translocation. However, in NSCLC there are many variants of the EML4–ALK fusion and many types of fusion partners, so this panel could miss ALK fusions that are not specifically included in the analysis. The efficacy of ALK‐TKI for PC with an ALK fusion gene is still unknown, but most reported cases suggest that ALK mutations in PC and PSC might be negative predictive factors of the efficacy of not only chemotherapy but also ALK‐TKI.^54^, ^55^

The *MET* exon‐14‐skipping mutation is considered one of the treatable driver mutations of NSCLC. The frequency of the *MET* exon‐14‐skipping mutation in pulmonary adenocarcinoma is about 3%.^56^ Several previous studies have identified high frequencies of the *MET* exon‐14‐skipping mutation (approximately 20%) in both PSC^34^, ^57^ and PC of the lung,^58^ which is higher than in adenocarcinoma. However, other studies have reported that the frequency of *MET* mutation is low.^36^, ^59^ In our study, two of the nine patients had a *MET* mutation, but not the exon‐14‐skipping mutation. In a case report, a *MET* inhibitor showed efficacy in the treatment of PSC with the *MET* exon‐14‐skipping mutation,^60^ but there have been too few reports to estimate the clinical effect of *MET* inhibitors.

A programmed cell death 1 (PD‐1) blockade was recently reported to elicit good responses in some patients with PC of the lung.^61^ PSC tumors have many somatic mutations, which is associated with the clinical efficacy of immune checkpoint inhibitors (ICIs).^62^ NSCLC with *SMARCA4* mutations displays a high mutation burden, so *SMARCA4* mutations are associated with the sensitivity of ICIs and are expected to be one of the predictive biomarkers of ICIs. Some patients with *SMARCA4*‐deficient tumors reportedly show a dramatic response to PD‐1 blockade.^63^ *SMARCA4* mutations are found in approximately 10% of NSCLC.^64^, ^65^ However, the frequency of *SMARCA4* mutations in PC of the lung is unknown. In the present study, we detected none of the *SMARCA4* mutations reported in the COSMIC database. Therefore, the association between the presence of *SMARCA4* mutations, a high tumor mutation burden, and the efficacy of ICIs for PC of the lung is still unclear.

This study had several limitations. First, we used an original gene panel containing 41 genes that are thought to be mutated frequently in lung cancer and to be biologically significant, which is fewer than can be detected with a commercial gene panel. Therefore, our panel may have missed some gene mutations related to the oncogenicity of PC of the lung. Second, the sample size was small, because this disease is rare and tissue availability consequently low. Consequently, the frequency of mutations may not have been accurately evaluated, and further extensive research is required. Finally, we did not evaluate the relationship between the detected gene mutations and the responsiveness of the tumors to candidate drugs or the clinical course of the disease. Therefore, the clinical value of the gene mutations detected in PC of the lung was not adequately evaluated.

In summary, we have demonstrated some driver‐gene mutations in PC of the lung. The driver‐gene mutation status of PC of the lung is still imprecise and controversial because it is a rare cancer. However, our data suggest that mutations in the *TP53* gene are frequent and co‐mutations are sometimes present in PC of the lung, as in some previous studies. Further intensive genomic NGS analyses based on these findings should identify the genomic characteristics of and treatment options for PC patients.

## Disclosure

All authors declare that they have no conflicts of interest.

## Supporting information


**Table S1** Original gene cancer panel, KCC41, including 41 genes thought to be associated with tumors of the lung.

**Table S2** Fusion variants of the *ALK*, *RET*, *ROS1*, and *NTRK1* genes from the Ion AmpliSeq RNA Fusion Lung Cancer Research Panel

**Table S3** NGS results for nine patients with pleomorphic carcinoma
